# Awareness and Practices of a Rural Community Regarding Mental Health Problems

**DOI:** 10.7759/cureus.40263

**Published:** 2023-06-11

**Authors:** Vivek Jayan, S Vishwas

**Affiliations:** 1 Epidemiology and Public Health, Sri Devaraj Urs Academy of Higher Education and Research, Kolar, IND

**Keywords:** stigma, rural, awareness, knowledge, mental health

## Abstract

Introduction: Mental health is defined as "a state of well-being in which the person realizes his or her own skills, can deal with the normal stresses of life, can work effectively and fruitfully, and is able to make a contribution to his or her community." Although mental health is essential to human survival, it is often given less attention than physical health in many parts of the world.

Objectives: The aim of this study is to evaluate the rural community's awareness regarding mental health issues and the factors that contribute to them.

Materials and methods: A cross-sectional study was undertaken in the rural community; 350 study subjects were selected from the village of Devarayasamudra by using convenient sampling, 350 households were selected, and household-level interviews were done using the Mental Health Knowledge Schedule questionnaire. Participants aged more than 18 were included in the study, and locked households, even after two visits, were excluded from the study.

Results: The median aggregate knowledge score was 31 (SD = 7.1), with the minimum and maximum values being 11 and 44 out of 45 knowledge items, respectively. The total knowledge score found that 178 (50.8%) respondents had insufficient mental health knowledge based on the percentage of the study population with a cut-off score below and above the median score. A multivariate logistic regression analysis confirmed that participants who were illiterate had 1.76 (1.15-2.26) times the chances of having insufficient knowledge compared to professionals, and this remained true even after adjusting for other variables as well.

Conclusion: The present study concluded that more than 50% (50.8%) of the participants had inadequate awareness of mental health and mental illness. This highlights the need to spread awareness about mental health education among the general community.

## Introduction

Mental health is defined as "a state of well-being in which the person realizes his or her own skills, can deal with the normal stresses of life, can work effectively and fruitfully, and is able to make a contribution to his or her community" [1]. Mental health is essential to human life; however, many countries prioritize physical health over mental health. This may be due to mental health stigma [2]. People's awareness of mental health issues is limited to those that are more severe or manifest later in the disease's progression. It's possible that this is due to the prevalence of common symptoms such as hopelessness and anxiety or that people are merely ignorant of their existence [3-6].

Suicide is widespread among those suffering from mental illnesses, and among those aged 15 to 29, it is the fourth leading cause of death. People with serious mental health disorders are expected to die up to 20 years sooner than the normal population for curable physical reasons [7]. It is generally acknowledged that informing the public about major bodily illness prevention, early intervention, and treatment will benefit them. Many individuals know that safe sexual behavior reduces HIV risk, smoking causes many diseases, and a balanced diet is essential. People usually recognize the early indications of cancer, heart attacks, and strokes and may have taken a first-aid course to learn how to treat these and other medical emergencies. Early intervention needs this information. In contrast, many people don't know what they can do to avoid mental illnesses, are distrustful of suggested treatments, and don't know how they can help those who are suffering [8].

Most people in the community would avoid talking to someone with a history of mental illness, and even fewer would consider befriending them [9]. Many studies have shown that people who are labeled as mentally ill are seen in a less positive light and are more likely to be rejected, no matter what they do [10].

It is important to enhance community awareness of mental health issues, the availability of effective treatments for mental illness, and how to detect and treat these illnesses. [6] The study of the community's awareness, attitude, and health-seeking behaviors in relation to mental illness may help in the provision of mental healthcare services [8].

The purpose of this study is to assess the levels of mental health awareness and practices in rural communities, as well as the variables associated with them. "This article was previously presented as a poster at the 5th Amrita International Public Health Conference in December 2022 at the Amrita Institute of Medical Sciences in Kochi."

## Materials and methods

Study design and settings

A cross-sectional method was used for this study, which was done in the rural regions of Devarayasamudra in the Kolar district of Karnataka, India. The study was conducted by visiting from house to house.

Study population

About 350 study participants were selected from the village of Devarayasamudra using a convenient sampling technique; 350 households were selected, and interviews with household members were conducted. Participants older than 18 were included in the study, and despite two visits, the locked household was excluded.

Study tools and measurements

To assess the sociodemographic profile, semi-structured questionnaires were used, and the adapted version of the Mental Health Knowledge Schedule (MAKS) [2] with a "yes" or "no" response was used to measure the community's knowledge of mental health problems. During the calculation of the median score for knowledge-based queries, a cut-off point below and above the median score was considered in order to determine the proportion of community members with abundant and insufficient knowledge.

Data analysis

The collected data were cleansed, coded, entered in Microsoft Excel (Microsoft, Washington, USA) and exported to SPSS Statistics version 22 (IBM Corp. Released 2013. IBM SPSS Statistics for Windows, Version 22.0. Armonk, NY: IBM Corp.) for analysis. To summarize the dependent and independent variables, descriptive statistics were conducted. Using the logistic regression analysis model, the factors associated with the outcome variable were identified.

Ethical clearance

Institutional ethical committee approval was obtained (No. DMC/KLR/IEC/392/2022-23).

## Results

Sociodemographic characteristics

A total of 350 study participants were successfully interviewed. The respondents' mean age was 44.36 (SD 16.9). The majority of participants in the study were men (193, 55.1%), married (271, 77%), and from nuclear families (223, 63%). Furthermore, according to the modified BG Prasad classification, the majority of the study participants belonged to the class 1 socioeconomic classification, and 309 of the participants were Hindu by religion. Furthermore, 30% of the participants only had a high school education (Table 1).

**Table 1:** Sociodemographic characteristics of participants

		Frequency	(%)
Sex	Male	193	55
	Female	157	45
Age	44.36 (SD ± 16.9)	-	-
Type of family	Nuclear family	223	63
	Joint/extended family	127	37
Socioeconomic classification (Modified BG Prasad 2021)	Class 1	118	33
	Class 2	94	27
	Class 3	74	21
	Class 4	43	12
	Class 5	18	5
Religion	Hindu	309	88
	Christian	19	5
	Others	22	7
Educational status	Unable to read and write	60	17
	Primary school	33	9
	Middle school	31	8
	High school	105	30
	Diploma	58	16
	Graduate	43	12
	Professional degree	20	5
Birth order	1^st^ child	160	46
	2^nd^ child	115	33
	3^rd^ child	52	14
	4^th^ child	7	2
	More than 4	14	5
Occupational status	Unemployed	60	17
	Unskilled	33	9
	Semiskilled	31	8
	Skilled	105	30
	Clerical/shop/farm	58	16
	Semi-professionals	43	12
	Professional	20	5
Marital Status	Married	271	77
	Unmarried	70	20
	Divorced	1	3
	Widowed	8	2

Respondents' mental health awareness

Nearly three-quarters (73%) of respondents know that mental illness is a type of medical disease, and nearly two-thirds (68.5%) know that mental health problems can be managed. In contrast, 31.7% of those surveyed believed mental illnesses were infectious, and 41.7% recommended isolation as a therapeutic option. In addition, over half of those polled (202 or 57.7%) disagreed that men and women experience the same kind of mental health issues. Participants listed aggression or violence (86%) and hearing or seeing objects that aren't there (83%) as major symptoms of mental illness (Table 2).

**Table 2:** Knowledge of the respondents regarding mental health and mental health problems

Variables	Characteristics	Frequency	Percentage
Mental illness is due to	Genetic reasons	173	49.4%
	Stress/tension	237	67.71%
	Accident/Injury	248	70.85%
	Brain functional abnormality	250	71.42%
	Family conflict	236	67.42%
	Conflict in family	227	64.85%
	Worrying much	240	68.57%
	Neurotransmitter imbalances	220	62.85%
	Witchcraft	169	48.28%
	God’s punishment for past sins	177	50.57%
	Possession of evil spirit	157	44.85%
	Personal weakness	155	44.28%
	Bad nutrition	182	52.00%
	Polluted atmosphere	131	37.42%
Mental illness can be treated	Traditional	100	28.57%
	Religious	96	27.42%
	Medical	286	81.71%
Professional counseling may help mental disease patients	-	283	80.85%
Mental disorders can be effectively treated with medication	-	312	89.14%
Mental illness requires psychiatric facility treatment	-	286	81.71%
Mental illness can be managed by families at home	-	205	58.57%
Psychiatric disorders should be treated by witch physicians	-	170	48.57%
Marriage can cure psychological disorders	-	199	56.85%
Free mental health care is freely available at all government health facilities	-	246	70.28%
Govt had dedicated counselors and support programs for mental illness	-	264	75.42%

Overall awareness level of the study participants

The median score for knowledge was 31, with the lowest score being 11 and the highest score being 44 out of 45 knowledge items. The total knowledge score revealed that 178 (50.8%) respondents had insufficient knowledge regarding mental health issues. This was determined by calculating the percentage of the study population that scored below and above the median level. Variables such as gender, age, degree of education, marital status, and employment were incorporated into the bivariate logistic regression analysis. With a P-value of less than 0.05, it was determined that education level and occupation were statistically significant determinants of mental health knowledge. Finally, multivariate logistic regression analysis confirmed that participants who were illiterate had 1.76 (1.15-2.26) times the chances of having insufficient knowledge compared to professionals, and this remained true even after adjusting for other variables as well. Even after controlling for confounding factors, those with a skilled occupational status had 1.87 times the chance of having insufficient knowledge as those with a professional level (Table 3).

**Table 3:** Factors related to mental health and mental health issues

Variables	Category	Knowledge of mental health problems		OR (CI)	AOR
		Inadequate	Adequate		
Sex	Male	92 (47.7%)	101 (52.3%)	0.752 (.493-1.147)	
	Female	86 (54.8%)	71 (45.2%)	1	
Age	18-31	46	75	1.12 (0.86- 1.75)	
	31-49	80	68	0.82 (0.48-1.13)	
	Above 50	52	29	1	
Marital status	Married	123	148	1.16 (0.60 -1.80)	
	Unmarried/widow/separated	55	24	1	
Level of education	Unable to read and write	44	16	1.76 (1.15-2.26)**	1.81 (1.28-2.38) **
	Primary school	7	26	1.46 (0.86- 1.98)	1.53 (0.89- 2.15)
	Middle school	23	8	0.89 (0.42- 1.26)	0.99 (0.62- 1.39
	High school	54	51	0.55 (0.36- 1.05)	0.72 (0.42- 1.20)
	Diploma	40	18	0.63 (0.33-0.96) **	0.46 (0.26-0.82) **
	Graduate	6	37	0.98 (0.67-1.16)	0.86 (0.47-1.09
	Professional degree	4	16	1	1
Occupational status	Unemployed	8	26	1.55 (0.89- 2.12)	1.44 (0.74-1.96)
	Unskilled	12	36	1.72 (1.05- 2.84) **	1.79 (1.12-2.75)**
	Semiskilled	14	20	1.62 (0.95-2.12)	1.48 (0.86-1.94)
	Skilled	16	11	1.87 (1.16-2.56) **	1.75 (1.05-2.28)**
	Clerical/shop	26	20	1.04 (0.86-1.68)	1.15 (0.96- 1.78)
	Semi-professionals	52	34	1.16 (0.84-1.48)	1.04 (0.75-1.57)
	Professional	50	25	1	1

## Discussion

More than 50% (50.8%) of the participants in the present study held only a fundamental understanding of mental health and mental illness.

Ganesh reported a similar kind of outcome in New Delhi, India, in 2011 by performing a cross-sectional study to measure the knowledge, attitude, and behavior surrounding mental disease and showing that general public awareness of mental illness was relatively inadequate [11]. Similarly, in Nigeria, Gureje et al. found that negative perceptions about mental illness were prevalent, with as many as 96.5% feeling that people with mental illnesses are hazardous due to their aggressive behaviors. Most people would not accept even basic social relations with a mentally ill person: 82.7% would be scared to talk to one, and only 16.9% would contemplate marrying one [12].

The present study observed that education level and occupation were statistically significant determinants of mental health knowledge, and the participants who were illiterate had 1.76 (1.15-2.26) times the odds of having insufficient knowledge compared to professionals; this was similar to another Nigerian study conducted by Kabir et al. among 250 adults that observed that literate respondents were seven times more likely to exhibit positive emotions toward the mentally ill as compared to non-literate subjects, and they also found that the most common symptoms proffered by respondents as manifestations of mental illness included aggression or destructiveness (22.0%) and loquaciousness (21.2%). About 46% of respondents favor standard medical care for the mentally ill, while 34% are more inclined toward spiritual healing [13].

In 2019, Abolfotouh et al. conducted a cross-sectional survey on 650 Saudi adults, and they found that the majority of the Saudi public reported a lack of knowledge about the nature of mental illness (87.5%), negative perception (59%), negative attitudes to mental illness (66.5%), and negative attitudes to professional help-seeking (54.5%) [14]. Although there is a significant prevalence of mental health disorders that affect every society in every region of the world, less focus and attention have been placed on finding effective solutions to close the knowledge gap, as evidenced by the general lack of information present across all research. This study suggests that the development of mental illness involves a complex interaction between biological, psychological, and cultural factors. In northern Ethiopia, Abbay et al. found that the level of mental health knowledge among the participants was low. They additionally found that factors such as being male, having a higher level of education, and having strong levels of social support were independent predictors of good mental health and community mental health knowledge, with both studies using the same questionnaire to assess the knowledge [15].

Furthermore, South African research uncovered religious and cultural explanations for the causes of mental illness [16]. The representation of the causes of mental illness in different countries may be different for a variety of reasons, including socioeconomic status, illiteracy rates, and the urban-rural study context. Respondents to the study almost unanimously agreed that chatting or laughing to oneself, as well as acting in a strange or unusual manner, are symptoms of mental illness [17]. The findings of research carried out in India are in agreement with this. Most of the people who answered this poll said they treated mental illness with a mix of medical, religious, and traditional methods. This is similar to the research done in Saudi Arabia [2]. Comparable to research conducted in New Zealand, nearly 80% of the participants in this study reported that mental illness is treatable [18].

The limitations of the study are that the cross-sectional structure of the data does not enable a rigorous causal interpretation of the findings. The present study assessed knowledge of mental illness by employing MAKS questionnaires, which were sensitive to memory bias. Although the study was representative of the general population, nonresponses may have had levels of knowledge and attitudes around mental illness and mental health help-seeking that were different from those of responders. It is possible, albeit difficult, to forecast how this may have impacted the results.

## Conclusions

The present study concluded that more than 50% (50.8%) of the participants had inadequate awareness of mental health and mental illness. This highlights the need to spread awareness about mental health education among the general community. Early identification of mental disorders, improved mental health outcomes, and increased use of health services may result from increased knowledge about mental health and mental disorders, better awareness of how to seek help and treatment, and reduced stigma against mental illness at the individual, community, and institutional levels.
